# Refractory immune thrombocytopenia: Lessons from immune dysregulation disorders

**DOI:** 10.3389/fmed.2022.986260

**Published:** 2022-09-20

**Authors:** Giorgio Costagliola, Rita Consolini

**Affiliations:** Section of Clinical and Laboratory Immunology, Division of Pediatrics, Department of Clinical and Experimental Medicine, University of Pisa, Pisa, Italy

**Keywords:** abatacept, autoimmune lymphoproliferative syndrome, eltrombopag, immunodeficiency, inborn errors of immunity, rituximab, sirolimus

## Introduction

Immune thrombocytopenia (ITP) is a heterogeneous condition that comprehends forms with acute presentation, persistent disease course (lasting for more than 3 months from disease onset), and chronic disease course, with a duration longer than 12 months (1). While the management of acute ITP relies mostly on the administration of intravenous immunoglobulin (IVIG) and corticosteroids (2), the second-line therapeutic options used in patients with persistent and chronic ITP are numerous, and comprehend biologic agents (particularly, the anti-CD20 monoclonal antibody rituximab), thrombopoietin receptor agonists (TPO-RAS), such as eltrombopag and romiplostim, and immunosuppressive agents (mycophenolate mofetil, azathioprine, and others) (2, 3). Differently from the past decades, splenectomy is currently performed only in a limited number of patients, on the basis of the individual risk-benefit assessment (4).

Agents active on the immune system and TPO-RAS could be either used alone or in combination regimens, and current guidelines for the management of ITP do not strictly regulate the choice of the second-line treatment or the possibility of combining different agents (3). Therefore, the approaches used in daily clinical practice are markedly heterogeneous.

Despite the wide number of therapeutic options, a small percentage of the patients with chronic ITP show refractoriness to treatment, which is defined by the lack of hematological response (defined by a platelet count < 30,000/μl or relapses with bleeding) to immunosuppressive agents and TPO-RAS (5). Notably, patients with refractory ITP have a poor response rate following splenectomy (5, 6). Therefore, there is an urgent need for new therapeutic strategies against refractory ITP.

The biology of refractory ITP is largely unexplored. It is primarily recommended to reconsider the diagnosis, as refractory ITP could be the first sign of rheumatic diseases (mostly, systemic lupus erythematosus) or inborn errors of immunity (IEI) (2, 7). Among IEI, there is increasing interest on a subcategory of diseases called “immune dysregulation disorders” (IDD), which includes autoimmune lymphoproliferative syndrome (ALPS) and related conditions, disorders of regulatory T cells (Tregs), combined immunodeficiencies associated with autoimmunity and lymphoproliferation, and others (8, 9). Interestingly, immune thrombocytopenia is the leading sign of immune dysregulation in many of these immune disorders. The knowledge of the molecular pathogenesis underlying their phenotypic picture led to the identification of targeted therapies whose adoption (10) has significantly improved the disease management of IDD disorders, resulting in effectiveness in the treatment of their hematological manifestations (8, 10).

Moreover, the experience gained from the treatment of IDD-associated autoimmune cytopenia could provide new therapeutic strategies even for patients with refractory ITP in whom the IDD diagnosed has been excluded.

## Immune dysregulation disorders associated with immune thrombocytopenia: From pathogenesis to treatment

Autoimmune lymphoproliferative syndrome is one of the better-characterized diseases among the group of IDD. It is an inherited disease caused by mutations impairing the first apoptosis signal (FAS) molecular pathway, and particularly the FAS, FAS ligand (FASL), and caspase-10 (CASP-10) genes, with about 30% of the patients remaining without a molecular diagnosis (11). The ineffective apoptosis is associated with the accumulation of αβ double-negative T CD8 + cells (abDNT) and self-reactive T cells (12). At disease onset, the most frequently observed manifestations are features of lymphoproliferation (lymphadenopathy, splenomegaly, and hepatomegaly) and autoimmune cytopenia, with ITP and autoimmune hemolytic anemia being the most common cytopenia (11, 13). Since the mammalian target of rapamycin (mTOR) molecular pathway is central to the proliferation and expansion of abDNTs, the use of sirolimus in patients diagnosed with ALPS is part of routine clinical practice. Sirolimus has demonstrated efficacy in the management of lymphoproliferative features and autoimmune cytopenia in patients with ALPS (14). Sirolimus is also successfully administered in other IEI with ALPS-like presentation, such as activated phosphoinositide 3-kinase δ syndrome (APDS), RAG deficiency, and also disorders of Tregs (Tregopathies) (15).

Immune dysregulation, polyendocrinopathy, enteropathy, and X-linked (IPEX) syndrome is the most widely nown disease among Tregopathies (16). It is caused by mutations impairing the function of forkhead box protein 3 (FoxP3) transcription factor, which is pivotal for the proliferation of Tregs (16). IPEX is featured by a clinical triad of autoimmune enteropathy, endocrinopathy (i.e., thyroiditis and type 1 diabetes), and early-onset severe eczematous dermatitis, but also other autoimmune features, such as autoimmune cytopenias, are frequently observed. Specifically, ITP is described in 5–10% of the patients diagnosed with IPEX (17).

Other Tregopathies associated with ITP include the cytotoxic T lymphocyte antigen-4 (CTLA-4) deficiency, lipopolysaccharide (LPS)-responsive, and beige-like anchor (LRBA) deficiency, and other disorders associated with mutations in signal transducer and activator of transcription (STAT) factors (16). The clinical phenotypes of CTLA-4 and LRBA deficiencies show significant clinical and pathogenic overlap. Indeed, CTLA-4 causes the reduction of the co-stimulatory molecules CD80 and CD86 on antigen-presenting cells, thus reducing the activation of T-dependent response, and LRBA acts through the inhibition of the lysosomal degradation of CTLA-4 (16, 18). Both diseases are associated with a complex phenotype that includes susceptibility to infections, autoimmunity, and lymphoproliferation. Autoimmune cytopenias (mostly ITP and autoimmune hemolytic anemia) are reported in about 70% of the patients with CTLA-4 and LRBA deficiency, often in association with lymphadenopathy and splenomegaly (19). The biologic agent abatacept, a fusion molecule containing the extracellular domain of CTLA-4, selectively enhances CTLA-4 dependent signaling and increases the CTLA-4/CD28 balance, thus reducing T cell activation. With the shared molecular pathogenesis between the two disorders, abatacept represents a valid therapeutic strategy in patients with LRBA deficiency (8, 20, 21). Literature data on the use of abatacept in these two conditions are still limited: a recent systematic review by Jamee et al. reported 15 cases of CTLA-4 deficiency and 60 patients with LRBA deficiency treated with abatacept, respectively (19).

Patients with disorders involving the JAK-STAT signaling pathways, and particularly STAT3 gain of function (GOF) and STAT1 GOF mutations, often present with an IPEX-like phenotype (16). The consequences of the molecular defect are better characterized for STAT3 GOF, in which patients show reduced proliferation of Tregs, decreased expression of CD25, and altered T helper type 17 cell proliferation, while the mechanism leading to altered Treg proliferation in STA1 GOF is less defined (22). In STAT3 GOF, more than 80% of the patients develop autoimmune cytopenia, with ITP being described in 50–60% of the patients (23). Differently, autoimmune cytopenia is less common (< 10%) in STAT1 GOF, in which the clinical spectrum is dominated by susceptibility to infections and endocrinopathy (24). Although the definitive treatment of STAT-related disorders is represented by hematopoietic stem cell transplantation (HSCT), the use of JAK inhibitors (ruxolitinib and baricitinib) has proven efficacy in the management of immune dysregulation in this condition (8, 25). However, the overall number of patients with STAT-related disorders treated with JAK inhibitors is still low. Indeed, the administration of JAK inhibitors has been reported only in 18 patients with STAT1 GOF and 13 patients with STAT3 GOF (27 receiving ruxolitinib, 3 tofactinib, and 1 baricitinib, respectively) with a high response rate but a reported short duration of follow-up (25).

Finally, as the activation of STAT3 is also triggered by interleukin (IL)-6, the anti-IL-6 antibody tocilizumab is a promising alternative for patients with severe disease and multisystemic involvement and its use has been described only in less than 10 patients with STAT3 GOF so far (25).

## Clinical implications and perspectives

The phenotypic spectrum of IDD widely includes thrombocytopenia as the main or leading, sometimes unique sign. This strongly highlights the need to investigate patients with refractory ITP for an underlying IDD. Furthermore, the evolving knowledge of the mechanisms of lymphocyte proliferation and apoptosis, Treg function, contributing to the molecular aspects responsible for the immune dysregulation of these patients, led to the search for targeted therapies directed against mTOR and JAK-STAT pathways, serum cytokines, and influencing CTLA-4-dependent events (Table 1). The increasing positive experience with these therapeutic strategies on the management of the hematological manifestations of patients with IDD carries significant implications, providing a rationale for the treatment of ITP refractory patients even in absence of a defined diagnosis of IDD.

**TABLE 1:** Immune dysregulation disorders associated with refractory immune thrombocytopenia (ITP) and targeted therapies.

Disease	Targeted therapy	References
ALPS	Sirolimus	14, 15
ALPS-like disorders	Sirolimus	15, 29
CTLA-4 deficiency	Abatacept	19
LRBA deficiency	Abatacept	19
STAT3 GOF	JAK inhibitors (ruxolitinib, baricitinib) Tocilizumab	8, 25
STAT1 GOF	JAK inhibitors (ruxolitinib, baricitinib)	8, 25

## Implications for differential diagnosis

The differential diagnostic work-up of ITP refractory patients includes the search for an immune dysregulatory disorder. If that is the case, these patients could benefit from a specific molecular targeted therapy. Therefore, it is strongly recommended to perform an accurate personal and familial anamnesis and to search for other features of immune dysregulation (other autoimmune disorders, lymphoproliferation, and severe eczema) or susceptibility to infections. Additionally, a first-level immunological assessment (serum immunoglobulin levels and lymphocyte subpopulations) is mandatory in all patients with refractory ITP. In patients with clinical or laboratory features suggestive for IDD, second-level investigations (extended lymphocyte phenotyping, antibody response, and autoantibodies) and finally genetic testing, contribute to the formulation of a definitive diagnosis of immune disorder and lays the rationale for the adoption of targeted therapies.

## Implications for treatment of refractory primary immune thrombocytopenia

There is evidence that dysregulation of the immune response, impaired Treg function, mTOR activation, and cytokine-dependent signaling participate in the complex pathogenic process leading to ITP also in patients without an underlying IDD (26–28). Therefore, drugs selectively targeting immune dysregulation could amplify the therapeutic armamentarium against ITP, particularly in refractory cases.

In this regard, the administration of sirolimus in patients with autoimmune cytopenia (such as ITP and Evans syndrome) has resulted in efficacy in different studies, and its use in the specific setting of refractory ITP has been analyzed recently with promising results (29–31). Sirolimus resulted in a complete response rate (CRR) of 40% and a partial response rate of 45% in a cohort of 86 patients described by Feng et al., with a higher likelihood to respond in patients younger than 40 years (30). Similarly, a multicentric study by Li et al. evidenced a long-term CRR of 45% and an overall response rate of 75% in a cohort of patients with refractory cytopenia (31). Overall, sirolimus has been well tolerated in the analyzed cohorts. These results strongly support the use of sirolimus as a therapeutic alternative in patients with refractory ITP. Notably, in one of the analyzed studies, the response to sirolimus was higher in patients showing other features of immune dysregulation associated with ITP (i.e., lymphoproliferation) (29), thus suggesting its application in patients with refractory ITP and high clinical suspicion of IDD, even in the absence of a defined molecular diagnosis.

On the other hand, the use of abatacept or JAK inhibitors in patients with refractory ITP, in the absence of demonstrated CTLA-4 or LRBA deficiency or STAT-associated disorders, has not yet been explored. Therefore, also considering their safety profile, it is reasonable to reserve these therapeutic options to patients who had undergone genetic analysis.

Conclusively, refractory ITP still represents a considerable therapeutic challenge in daily clinical practice. Recognizing in these patients of an underlying IDD could provide the rationale for the adoption of targeted treatments. Moreover, the knowledge of the clinical spectrum and molecular pathogenetic mechanisms underlying ITP in IDD, together with the increasing experience gained with drugs targeting immune dysregulation, could open new perspectives for the treatment of patients with refractory primary ITP.

## Author contributions

GC and RC conceptualized the manuscript. GC drafted the manuscript. RC critically revised the manuscript. Both authors contributed to the article and approved the submitted version.
